# P14 Study of Prescribing patterns and Effectiveness of Ceftolozane/Tazobactam Real-world Analysis (SPECTRA): results from a multinational, multicentre observational study

**DOI:** 10.1093/jacamr/dlad143.018

**Published:** 2024-01-03

**Authors:** Alejandro Soriano, Laura Puzniak, David L Paterson, Florian Thalhalmmer, Stefan Kluge, Pierluigi Viale, Alexandre H Watanabe, Engels N Obi, Sunny Kaul

**Affiliations:** Hospital Clinic, Barcelona, Spain; Merck & Co. Inc., Rahway, NJ, USA; The University of Queensland, Queensland, Australia; Medizinische Universität Wien, Spitalgasse, Austria; Martinistrasse, Hamburg, Germany; Università di Bologna – Via Zamboni, Bologna, Italy; Merck & Co. Inc., Rahway, NJ, USA; Merck & Co. Inc., Rahway, NJ, USA; Royal Brompton & Harefield NHS Foundation Trust, London, UK

## Abstract

**Background:**

Ceftolozane/tazobactam (C/T) has demonstrated efficacy to treat complicated intra-abdominal infections (cIAI), complicated urinary tract infections (cUTI) and hospital acquired bacterial and ventilator-associated bacterial pneumonia. However, physicians, providers, and other stakeholders including payers want broader real-world evidence to inform clinical decisions and optimize healthcare resource use.

**Methods:**

SPECTRA is a multinational, multicentre, retrospective, inpatient, observational study of patients treated with C/T in Australia, Austria, Germany, Italy, Mexico, Spain and the UK. Adult inpatients treated with ≥48 h of C/T were included. Demographics, clinical characteristics, treatment management patterns, and outcomes were analysed.

**Results:**

There were 687 patients from 38 participating hospitals in 7 countries. The average age was 57.6 years (SD ±17.3), and most were male (456, 66.4%). The majority had at least one comorbidity (563, 82.0%), with the most common being heart disease (208, 30.3%), immunocompromised state (207, 30.1%) and chronic pulmonary disease (195, 28.4%). The most common indications were pneumonia (204, 29.7%), sepsis (147, 21.4%) and cIAI (106, 15.4%); 162 (23.6%) had multiple sites of infection and 245 (35.7%) were polymicrobial infections. Median C/T treatment was 12.0 days (IQR 11.0). Half of the patients were admitted to the ICU (343, 49.9%), 43.4% of which was related to the infection. Clinical success was 66.1%. All-cause in-hospital mortality was 22.0% with 8.7% being infection related. The 30 day all-cause readmission was 9.8% and 4.7% were infection related.

**Conclusions:**

C/T was used to treat infections among critically ill patients and for multi-source, polymicrobial infections. Despite the complexity of the patients in this real-world analysis, most C/T patients had beneficial outcomes that are similar to results of controlled clinical trials.

